# CyanOmics: an integrated database of omics for the model cyanobacterium *Synechococcus* sp. PCC 7002

**DOI:** 10.1093/database/bau127

**Published:** 2015-01-28

**Authors:** Yaohua Yang, Jie Feng, Tao Li, Feng Ge, Jindong Zhao

**Affiliations:** ^1^Key Laboratory of Algal Biology, Institute of Hydrobiology, Chinese Academy of Sciences, Wuhan 430072, China, ^2^University of Chinese Academy of Sciences, Beijing 100049, China, ^3^College of Life Science, Peking University, Beijing 100871, China

## Abstract

Cyanobacteria are an important group of organisms that carry out oxygenic photosynthesis and play vital roles in both the carbon and nitrogen cycles of the Earth. The annotated genome of *Synechococcus* sp. PCC 7002, as an ideal model cyanobacterium, is available. A series of transcriptomic and proteomic studies of *Synechococcus* sp. PCC 7002 cells grown under different conditions have been reported. However, no database of such integrated omics studies has been constructed. Here we present CyanOmics, a database based on the results of *Synechococcus* sp. PCC 7002 omics studies. CyanOmics comprises one genomic dataset, 29 transcriptomic datasets and one proteomic dataset and should prove useful for systematic and comprehensive analysis of all those data. Powerful browsing and searching tools are integrated to help users directly access information of interest with enhanced visualization of the analytical results. Furthermore, Blast is included for sequence-based similarity searching and Cluster 3.0, as well as the R hclust function is provided for cluster analyses, to increase CyanOmics’s usefulness. To the best of our knowledge, it is the first integrated omics analysis database for cyanobacteria. This database should further understanding of the transcriptional patterns, and proteomic profiling of *Synechococcus* sp. PCC 7002 and other cyanobacteria. Additionally, the entire database framework is applicable to any sequenced prokaryotic genome and could be applied to other integrated omics analysis projects.

**Database URL**: http://lag.ihb.ac.cn/cyanomics

## Introduction

Cyanobacteria were the first organisms to carry out oxygenic photosynthesis on the Earth; this photosynthetic capability changed the status of the atmosphere, and they continue to play important roles in the Earth’s carbon and nitrogen cycles (1). Cyanobacteria also possess a high potential for biofuel production, and many valuable co-products can be obtained during the same processes (2). *Synechococcus* sp. PCC 7002 (hereafter *Synechococcus* 7002) is a unicellular cyanobacterium that grows well under a wide range of sodium chloride concentrations (3). It can survive high-light intensities and many other extreme conditions (4, 5) and is regarded as an ideal model cyanobacterium. *Synechococcus* 7002 gene expression has been studied under various conditions, which greatly contributes to our understanding of its functional genomics and potential biotechnological applications (6).

The complete genome sequence (including plasmids) of *Synechococcus* 7002 is available (http://www.ncbi.nlm.nih.gov/nuccore/) and has been fully annotated. With the rapid development of next-generation sequencing technologies, a number of *Synechococcus* 7002 transciptomes of cells grown under normal and perturbed conditions have been generated during the past several years, including exposure to high-light and dark incubation (7), nutrient (CO_2_, nitrogen, sulfate, phosphate and iron) limitation and different kinds of nitrogen sources (8), different temperatures and salinities and mixotrophic conditions (9, 10). Several proteomes of *Synechococcus* 7002 grown under different conditions have also been determined (11, 12). These studies undoubtedly provide us with massive and openly accessible transcriptomic and proteomic information on transcriptional regulation and gene expression patterns. However, different transcriptomes and proteomes corresponding to different conditions were not integrated in those reports, and they were deposited in individual repositories. So far, no systematic and comprehensive analysis platform is available for researchers to perform bioinformatics studies on those datasets, and the great potential of the massive amount of data has not been realized. Therefore, an urgent need exists for an integrated analysis database that can produce direct and comprehensive analysis results across various studies. This should prove to be a huge convenience for experimental biologists.

Several cyanobacteria databases presently exist, such as Cyanobase (13), SynechoNET (14) and CyanoEXpress (15). However, none of these are specially designed and built for the massive and powerful omics datasets. Here we present CyanOmics, the first integrated omics database for cyanobacterium. CyanOmics is comprised of the complete genome sequence with functional annotation, transcriptomes under standard and different perturbed conditions, and proteomic analyses of *Synechococcus* 7002, integrating functions for browsing, navigating, sequence alignment and data visualization. It aims to provide a comprehensive and systematic analysis platform for cyanobacteriologists worldwide. An overview of the entire catalogue of the gene annotation information, gene expression abundances under specific conditions and proteomic profiling can be obtained through CyanOmics. Furthermore, the relative abundances of gene expression over all the conditions can be displayed and compared, which can help researchers with experimental design to further validate the data. Users can also perform personalized analyses with the built-in tools in CyanOmics according to their own requirements, greatly facilitating applications of the database.

## Database construction

The complete genome sequence of *Synechococcus* 7002 (3 Mb) and sequences of six plasmids (4.8, 16, 32, 39, 124 and 186 Kb) were downloaded from the National Center for Biotechnology Information (NCBI) Nucleotide database (http://www.ncbi.nlm.nih.gov/nuccore/) with accession numbers: NC_010475.1, NC_010476.1, NC_010477.1, NC_010478.1, NC_010479.1, NC_010480.1 and NC_010474.1. All of the *Synechococcus* 7002 transcriptomes from the Bryant lab (7-9) were downloaded from the NCBI Sequence Read Archive (SRA) (16) with accession numbers: SRP004049, SRP007372 and SRP013965. The SRA format datasets were converted to fastq format using the NCBI SRA Toolkit (http://www.ncbi.nlm.nih.gov/Traces/sra/?view=software), and then were filtered with a Perl script (Q20 standard) to eliminate low-quality reads. The filtered high-quality transcriptomic reads from each growth condition were aligned against the *Synechococcus* 7002 complete genome sequence individually using Burrows-Wheeler Aligner (BWA) software (17) with a seed sequence length of 15. Sequences that did not map to the reference genome in this step were eliminated. To get the unique mRNA from protein-coding regions, the reads which were mapped to the rRNA-coding regions and aligned to more than one region over the genome sequence were removed from the BWA-mapped alignment results. The remaining alignments in SAM format were converted to BAM format files and sorted in the same order as the names of the chromosomes and plasmids with SAMtools (18). Next, the reads assigned per kilobase of target per million mapped reads (RPKMs) (19), and fold changes (relative to the standard samples in the corresponding experiment) for uniquely mapped genes in each individual transcriptome were calculated and stored in a table. The calculation was carried out with in-house python scripts which are provided on the download page of CyanOmics web site. The χ^2^ test was performed on a gene-by-gene basis, to determine the differentially expressed genes for each of the conditions, and those genes with fold changes ≥2 or ≤−2 and *P* values < 0.05 were considered significantly up-regulated or down-regulated, respectively (20). The transcript levels of all the genes and *P* values under different conditions are provided in the Download module in the CyanOmics database. Detailed information about all of the growth conditions, including medium composition, light intensity, aeration state, temperature, growth time, harvest OD and culture environment status, was extracted from the related research literature and integrated one by one for each record in easily understandable text. Furthermore, we’ve integrated our recalculated and Bryant’s fold change results, on which the cluster analysis is performed for comparison. The cluster results indicate that the Bryant’s and our recalculated fold change data from the same growth conditions are clustered together, which means that the data reported in literatures and recalculated by us are in correspondence. The integrated fold change table and the cluster heatmap image are provided on the Download page in CyanOmics. The *Synechococcus* 7002 global phosphoproteome dataset was obtained from (11), from which 247 detected phosphorylated proteins were extracted. Relevant important information regarding those phosphorylated proteins, such as their peptide sequences, functional descriptions, phosphorylated amino acid sites [mainly Serine (S), Threonine (T) and Tyrosine (Y)], scores and search engine (pFind, Mascot), were integrated into a table. The Clusters of Orthologous Group (COG) functional categories (21) (including 22 categories from PCC 7002) were downloaded from the NCBI (http://www.ncbi.nlm.nih.gov/COG/) and were updated in 2013. CyanoBase gene functional categories were obtained from CyanoBase (http://genome.microbedb.jp/cyanobase/SYNPCC7002) and were updated in 2008. All the datasets used are listed in Table 1.

**Table 1.** List of the data sets applied in CyanOmics

	Accession no.	Description
Genome	NC_010475.1	Chromosome
	NC_010476.1	Plasmid pAQ1
	NC_010477.1	Plasmid pAQ3
	NC_010478.1	Plasmid pAQ4
	NC_010479.1	Plasmid pAQ5
	NC_010480.1	Plasmid pAQ6
	NC_010474.1	Plasmid pAQ7

Transcriptomes^a^	SRR097642	Dark oxic
	SRR097643	Dark anoxic
	SRR097644	High light
	SRR097645	Standard 1
	SRR097646	Standard 2
	SRR097647	Standard 3
	SRR097648	OD 0.4
	SRR097649	OD 1.0
	SRR097650	OD 3.0
	SRR097651	OD 5.0
	SRR097656	Low O_2_
	SRR308187	Low CO_2_
	SRR308188	N-limited
	SRR308189	S-limited
	SRR308190	P-limited
	SRR308191	Fe-limited
	SRR308192	Nitrate
	SRR308193	Ammonia
	SRR308194	Urea
	SRR1057531	Heat shock
	SRR1057994	22 °C
	SRR1057995	30 °C
	SRR1057996	Oxidative stress
	SRR1057997	Mixotrophic
	SRR1057998	Standard 1
	SRR1057999	Standard 2
	SRR1058000	Standard 3
	SRR1058001	Low salt
	SRR1058002	High salt

Proteomes	PASS00119 (PeptideAtlas)	Phosphorylation

^a^Descriptions of the transcriptomes correspond to their relative conditions (perturbations or standard condition).

**Figure 1.**
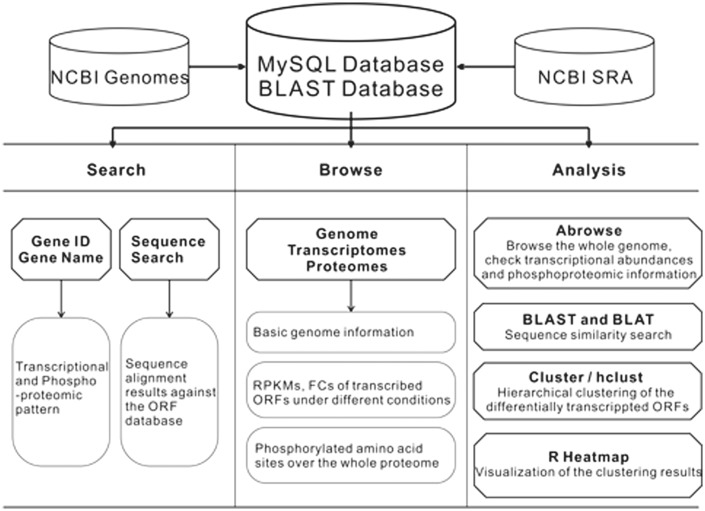
The CyanOmics scheme illustrating the datasets and analysis methodologies integrated in the database.

The schema of the CyanOmics database is shown in Figure 1. All the aforementioned genome, transcriptome, proteome and descriptive data were organized in a MySQL database. The web interface for analysing these data was written using the Python django framework. Apache2 (http://httpd.apache.org/) in a CENTOS operating system was used as the web server. The search functions made it easy to access the three major resources in CyanOmics. Abrowse (22) (http://www.abrowse.org) was incorporated to provide a convenient way to access the omics datasets using a graphical viewer and a built-in Blast-like alignment tool (BLAT) server. The NCBI Basic Local Alignment Search Tool (BLAST) program (23) was integrated into the CyanOmics web interface to provide sequence-based similarity searches against the formatted *Synechococcus* 7002 gene database. Cluster 3.0 (24) (http://bonsai.hgc.jp/∼mdehoon/software/cluster) was used for the cluster analysis between the transcriptomes under a series of different conditions or among the whole set of open reading frames (ORFs) under a specific condition. Hierarchical clustering is also provided by the hclust function from the R package (http://www.r-project.org/), which is able to visualize the analysis results as the calculation procedure is completed. Users can choose either cluster analysis method based on their preference.

## Database description and utility

### Web interface and homepage

The CyanOmics database interface provides direct links to six individual pages: Home, Genome Browser, Transcriptomics, Proteomics, Tools and Download. A detailed help document is also provided, which describes how to browse, search, analyse data and visualize results in each main page. All the links are clickable icons. A single click leads to the respective page.

**Figure 2.**
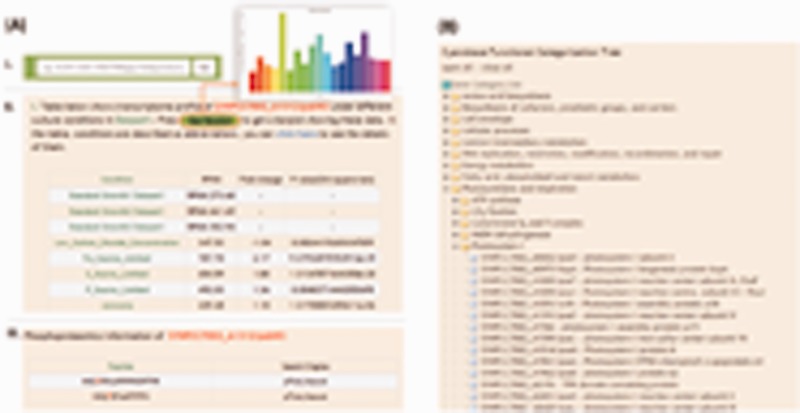
The search module and Cyanobase functional categorization tree integrated into the CyanOmics interface. (A) The search box and the extended search results page; (B) structure of the Cyanobase functional categorization tree on the website and an illustration of the drop-down list of the genes belonging to the corresponding category.

A search box is built into the top right of each page in the CyanOmics database so that users can search the database using a *Synechococcus* 7002 locus_tag or gene symbol (Figure 2A), helping users acquire information they are interested in from any dataset directly and quickly. The Search module supports fuzzy queries, e.g. ‘SYNPCC7002_A1312’ or ‘A1312’ can be input to find all the genomic, transcriptomic and proteomic information about the ORF ‘SYNPCC7002_A1312’, which functions as Photosystem II complex subunit. Users can also input a full or partial gene name, e.g. ‘psb’ to search for a series of genes involved in Photosystem II, such as psbA, psbU, psbO, psbD, pcbC, psbW2, etc. Gene product and NCBI gi ID are supported as keyword too, effectively broadening the search scope. The search results page includes both the transcriptomic and proteomic information related to the query object (Figure 2A). Users can launch the Genome Browser through the Gene Name hyperlink at the top of the results page, to further investigate the target ORF location, transcriptional abundances under varying conditions and proteomic identification information. Tables containing RPKM and fold change data of the target ORF are presented, and bar plot images can be seen by pressing a ‘barplot’ button above the table (Figure 2A). The bar plot image can be downloaded with a right click, ‘Save Image As’ option. Proteomics results are shown at the bottom of the results page, and include phosphorylation status and proteomic identification as a whole (Figure 2A).

A brief introduction to cyanobacterium *Synechococcus* 7002 and CyanOmics is included at the top part of the Homepage. Below this, a functional categorization tree is displayed, which can help users to access information of interest according to functional subsystems (Figure 2B). The functional categorization is obtained from the gene functional category of CyanoBase (http://genome.microbedb.jp/cyanobase/SYNPCC7002) and was updated in 2008. A single click on each functional category opens a drop-down list of ORFs belonging to that specific category. Clicking on any ORF produces a results page similar to that displayed by the search module (Figure 2A), i.e. a Genome Browser view, and transcriptomic and proteomic information. The left panel on the Homepage contains the latest *Synechococcus* 7002 research findings, several relevant important external links, and our contact information.

### Genome Browser

**Figure 3.**
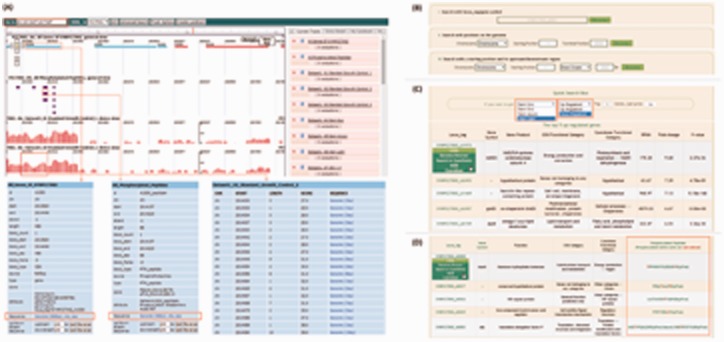
Screenshots of the Genome Browser, transcriptomics and proteomics interfaces. (A) Overview of the Genome Browser and an illustration of its application in browsing of genome, transcriptomes and proteomes; (B) three strategies to quickly search regions of interest using the Genome Browser; (C) check box of up- or down-regulated genes under different conditions, and information table of the differentially transcribed genes, including biological functions, Cyanobase functional categories, COG categories, fold changes and external links; (D) an information table of proteomic data.

A single click on the red ‘PCC 7002 Omics Browser’ button below the introduction of Abrowse on the Genome Browse main page opens a new browser window comprised of all the dataset tracks integrated into the CyanOmics database. An overview of the histogram navigator—Abrowse-mode Genome Browser—is shown in Figure 3A.

The window is divided into two parts. The relatively small panel on the right is a list of the datasets, on which the tags, from top to bottom, are: ‘All Genes of SYNPCC7002’, ‘All Phosphorylated Peptides’, ‘Standard Growth’ and the other 23 growth conditions. Clicking on the red ‘cross’ in front of the titles hides the corresponding tracks to help users focus on the tracks they are interested in more clearly and closely. Dragging the blue ‘two-way arrow’ up or down will change the track position to display datasets of interest at the top for convenience. The larger window on the left is the track display region and all the tracks are scalable and movable. Users can input a range into the ‘Go to’ box red marked in Figure 3A to locate a particular region of interest on the genome, or users can simply drag forward or backward along the tracks to achieve the same goal. Zooming in and out can be done with the ‘plus’ and ‘minus’ buttons at the top left. In the genomic view, rectangular frames represent ORFs with directionality: red ones in the forward direction while blue ones read backwards. Choosing an ORF by clicking it will display detailed information about that particular ORF in the right tab; the ‘Sequence’ heading (Figure 3A) allows nucleotide and amino acid sequences to be displayed in a new window. Purple rectangular frames represent phosphorylated regions in the phosphoproteomic view. A single click on the frame will open an information table similar to the table displayed in the genomic view (Figure 3A). The transcriptomic datasets are visualized as tracks with coverage-related red histograms. Coverage of each nucleic acid is represented by the height of the corresponding column: the higher the column, the denser the single nucleic acid is covered. Because coverages between different positions varied greatly (from one to hundreds of thousands), the absolute coverage of each bp was subjected to log transformation, the largest coverage of the dataset as the base, to improve the visualization. Clicking to select a fragment will open a new tab in the ‘Entry Detail’ region (Figure 3A), showing detailed coverage statistics. The score in the panel represents 100 times the value after log transformation. As with the genomic track, nucleotide sequences can be viewed through the ‘Sequence’ heading. All the genomic, transcriptomic, and proteomic navigators appear in one window, and users can easily and immediately gain a global impression of the datasets.

A multi-functional search module is integrated into the lower portion of the Genome Browser main page to facilitate users’ convenience in reaching specific regions of interest, (Figure 3B), largely increasing the browsing efficiency. Three search strategies are available: (i) Search with a gene ID or gene tag, supports fuzzy queries and is case insensitive. (ii) Search with starting and ending positions on the chromosome or a plasmid to quickly locate target regions. Each value of the positions must be an integer. Pressing on the ‘Browse’ button will lead to a display of the region on all of the dataset tracks. (iii) Search with a starting position and its adjacent fragment. Sometimes users want to examine the adjacent regions, upstream or downstream, of a known position, and this search strategy works for them. As with specifying beginning and ending coordinates, simply inputting the position and pressing the ‘Browse’ button displays the region of interest.

### Browsing data through transcriptomics and proteomics

A brief introduction to the *Synechococcus* 7002 RNA-seq datasets integrated into CyanOmics and links to the appropriated references are provided on the Transcriptomics main page. In transcriptomic studies, differentially expressed (up-regulated and down-regulated) genes are of great importance and are key points for researchers. Therefore, check boxes to obtain data related to these types of genes by clicking on the ‘Link to Dataset1’ or ‘Link to Dataset2’ buttons [Dataset1 contains transcriptomes from (7, 8) and Dataset2 contains transcriptomes from (9); Figure 3C]. Users can select any growth condition, or up- or down-regulated pattern, and can input an integer that represents the number of most highly regulated genes to be shown. Pressing the ‘Go’ button opens a new page, of which the upper part is a summary of the transcriptional landscape under the selected condition. The lower part is a table containing RPKM/fold change and functional category information for the genes selected. When hovering the mouse over the locas_tag in the first column, a list of links to Genome Browser, Search in CyanOmics, NCBI and CyanoBase databases appears; users can choose any of these to access the corresponding page (Figure 3C). For example, choosing the NCBI or CyanoBase links for SYNPCC7002_A1973/ndhD2, information about ndhD2 in these two databases will be shown in a new window. Choosing Genome Browser will switch to the Abrowse-mode display of the ndhD2-related information, while Search in CyanOmics will lead to the search results page as shown in Figure 2A. Below the quick search box are descriptions of the experimental conditions extracted from the literature sources, through which users can obtain a closer look into the relevant growth conditions.

The top part of the Proteomics main page contains an introduction to proteomics and the data we integrated into the CyanOmics database. Below is a text describing the experimental conditions, analysis procedures, and several methods provided by CyanOmics to access the proteomic datasets. Clicking on the hyperlink ‘table of all phosphorylated proteins and their detailed information’ opens a new window that contains Locus-tags of all phosphorylated genes, phosphorylated peptides and their corresponding biological functions (Figure 3D). Phosphorylated amino acids are highlighted in the table with red-marked letters. A list of links to our Genome Browser, Search in CyanOmics, NCBI and CyanoBase databases will appear when hovering the mouse over the Locus-tags in the first column, similar to what is described in the Transcriptomics section, and as shown in Figure 3C.

As stated earlier, the Transcriptomics and Proteomics sections are internally connected and can link to the Genome Browser, the search module and various external links.

### Tools

BLAST is integrated into the Tools section. Users can choose blastn or blastp program to apply in the ‘Basic Settings’ part, and the formatted reference databases are nucleic acid and amino acid sequences of ‘all genes of PCC 7002’. Nucleotide and protein sequences in FASTA format are accepted in the query panel or users can upload a local sequence file in FASTA format. Other standard BLAST parameters, such as E-value, alignment view mode and matrix are also provided. Clicking on the hyperlinks of object genes in the alignment results window opens a search results page as shown in Figure 2A, convenient for users to examine query-related transcriptomic patterns under different conditions and proteomic information. We have provided a ‘Run WWWBlast’ option, with which visualization of the resulting alignments would be obtained on the results page.

**Figure 4.**
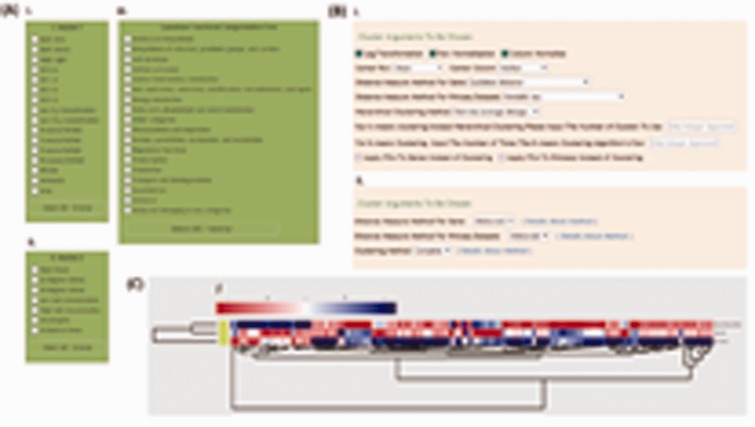
Screenshots showing the interfaces and the resulting cluster analysis images from Cluster 3.0 and hclust in the Tools section. (A) Three dataset selection boxes containing experimental conditions and functional categories in Cluster 3.0 and hclust; (B) parameter panels provided by Cluster 3.0 and hclust; (C) a sample heatmap of the cluster analysis results.

Users can choose the data on which to perform cluster analysis using the Cluster 3.0 software page based on two aspects: experimental conditions and functional categories (Figure 4A). This considerably narrows down the analysis range, and greatly shortens the analysis time, allowing users to be able to focus on information of interest, instead of browsing through all kinds of irrelevant data. The full set of Cluster 3.0 parameters is provided below the data selection table, including normalization, distance measure method and hierarchical clustering method (Figure 4B). Pressing the ‘Run Cluster’ button at the bottom of the page starts the calculation process, resulting in three cluster output files that contain detailed statistical information. What the items in the output files mean is explained on the hyperlink page ‘detailed explanation for output files’. A single click on the ‘Get Heatmap Image’ button will open a heatmap showing the up- and down-regulated patterns and clustering state of the genes and conditions selected (Figure 4C). The cluster image can be downloaded with a right click save-as option. The parameters on the Cluster 3.0 main page are clickable and a single click on each of them will open a page containing descriptions for corresponding statistics used in Cluster.

Another way to do cluster analysis is with the hierarchical clustering (hclust) function from the R package. The data selection tables are the same as applied with Cluster 3.0 (Figure 4A). ‘Distance measure method’ parameters for genes and datasets can be freely selected. Also users can choose which cluster method to employ through a drop-down list. Clicking the hyperlink ‘details about method’ provides an introduction to the cluster methods in CyanOmics (Figure 4B). Pressing the ‘Get Heatmap Image’ button on the results page opens a heatmap, the same as that produced by Cluster 3.0 (Figure 4C).

## Summary and future development

In summary, CyanOmics is the very first integrated omics database for the model cyanobacterium *Synechococcus* 7002, and for all cyanobacteria. It is comprised of useful information about the complete genome sequence, transcriptomic profiling and proteomic analyses and provides a comprehensive and systematic omics analysis platform for researchers to browse data of interest and make good use of them. CyanOmics contains a user-friendly interface, a well-designed database structure, enhanced visualization tools, and it provides several convenient operations, all easy for researchers to use, even those with little knowledge of bioinformatics. The Genome Browser, Transcriptomics and Proteomics sections are dynamically connected and can link to each other internally.

As *Synechococcus* 7002 is an important model organism for analysing physiological characteristics, photosynthesis and acclimation patterns of cyanobacteria under extreme environmental habitats (4, 5, 25-27), and especially with the rapid development of DNA sequencing technologies (next-generation and third-generation sequencing), and protein analysis methods (e.g. mass spectrometry technology), the number of transcriptomic, proteomic and other omics studies, such as epigenomic studies (methylomes, acetylomes) over the whole genome, will continue to increase in the future. CyanOmics will be periodically updated to stay in concert with the progress in omics studies of *Synechococcus* 7002.

To provide a wider view over all cyanobacteria, omics data of other model organisms, including *Synechococcus elongates* PCC 7942 (28), *Synechocystis* sp. strain PCC 6803 (29) and *Anabaena* sp. strain 7120 (30), will be added to CyanOmics in the future. Data management and analysis procedures will be the same as those applied on *Synechococcus* 7002 data, making modifications only as necessary. Comparative omics analyses among the different cyanobacterial genomes, transcriptomes, proteomes and epigenetic genomes will be performed and should provide much useful information.
